# Effectiveness of an Ambient Assisted Living (HomeAssist) Platform for Supporting Aging in Place of Older Adults With Frailty: Protocol for a Quasi-Experimental Study

**DOI:** 10.2196/33351

**Published:** 2022-10-26

**Authors:** Hélène Sauzéon, Arlette Edjolo, Hélène Amieva, Charles Consel, Karine Pérès

**Affiliations:** 1 Flowers Team Inria University of Bordeaux Talence France; 2 Bordeaux Population Health Center - U1219 Inserm University of Bordeaux Bordeaux France; 3 Bordeaux-INP Bordeaux France

**Keywords:** ambient assisted living technology, AAL, Internet-of-Things, IoT, aging and frailty, independent living, effectiveness study

## Abstract

**Background:**

Ambient assisted living (AAL) technologies are viewed as a promising way to prolong aging in place, particularly when they are designed as closely as possible to the needs of the end users. However, very few evidence-based results have been provided to support its real value, notably for frail older adults who have a high risk of autonomy loss as well as entering a nursing home.

**Objective:**

We hypothesized that the benefit from an AAL with a user-centered design is effective for aging in place for frail older adults in terms of everyday functioning (instrumental activities of daily-life scale). In addition, our secondary hypotheses are that such an AAL decreases or neutralizes the frailty process and reduces the rates of institutionalization and hospitalization and that it improves the psychosocial health of participants and their caregivers when compared with the control condition. We also assume that a large proportion of equipped participants will have a satisfactory experience and will accept a subscription to an internet connection to prolong their participation.

**Methods:**

HomeAssist (HA) is an AAL platform offering a large set of apps for 3 main age-related need domains (activities of daily-living, safety, and social participation), relying on a basic set of entities (sensors, actuators, tablets, etc). The HA intervention involves monitoring based on assistive services to support activities related to independent living at home. The study design is quasi-experimental with a duration of 12 months, optionally extensible to 24 months. Follow-up assessments occurred at 0, 12, and 24 months. The primary outcome measures are related to everyday functioning. Secondary outcome measures include indices of frailty, cognitive functioning, and psychosocial health of the participants and their caregivers. Every 6 months, user experience and attitudes toward HA are also collected from equipped participants. Concomitantly, data on HA use will be collected. All measures of the study will be tested based on an intention-to-treat approach using a 2-tailed level of significance set at α=.05, concerning our primary and secondary efficacy outcomes.

**Results:**

Descriptive analyses were conducted to characterize the recruited equipped participants compared with the others (excluded and refusals) on the data available at the eligibility visit, to describe the characteristics of the recruited sample at baseline, as well as those of the dropouts. Finally, recruitment at 12 months included equipped participants (n=73), matched with control participants (n=474, from pre-existing cohorts). The results of this study will be disseminated through scientific publications and conferences. This will provide a solid basis for the creation of a start-up to market the technology.

**Conclusions:**

This trial will inform the real-life efficacy of HA in prolonging aging in place for frail older adults and yield an informed analysis of AAL use and adoption in frail older individuals.

**International Registered Report Identifier (IRRID):**

DERR1-10.2196/33351

## Introduction

### Background

Frailty is a common geriatric syndrome characterized by age-related declines in both physical and cognitive reserves, as well as physiological function, leading to increased vulnerability to adverse health outcomes (eg, [1]). According to the physical phenotype proposed by Fried et al [2], frailty refers to individuals meeting at least three of the following 5 criteria: weakness, slowness, low level of physical activity, self-reported exhaustion, and unintentional weight loss. In the community-dwelling older population, frail and prefrail individuals represent approximately 40% of people aged >65 years [3]. Frail individuals (FI) are at a higher risk of disability, hospitalization, and institutionalization [4,5] and are recognized as an optimal target population for the implementation of effective programs to prevent dependency [3] or even to reach a successful aging path [6]. As highlighted by the World Health Organization in its healthy aging concept, environments and their interactions with intrinsic capabilities play a substantial role in developing or maintaining functional abilities that enable well-being in older age [6]. In this FI population, environmental support can be a relevant approach to encourage and facilitate the instrumental activities of daily life (IADLs). In turn, this contributes to reducing or delaying functional degradation and fostering and extending aging under good conditions [7].

Assistive technologies (ATs) for activities of daily life (ADLs) refer to all technical forms of environmental support that provide an adaptation of the environment to make it more accommodating for persons with impairments [8]. Despite enthusiasm for ATs, several issues remain to be resolved. First, all the studies published so far on AT lack empirical evidence of their efficacy, mostly because of shortcomings related to study design (small sample size, irrelevant or nonstandardized measures, short follow-up duration, no control group, etc) [9,10]. Second, despite the many technological innovations available to assist older adults in their daily life [9], their silo-based nature (one technology per need) makes them challenging to integrate, as older adults require more services to assist an increasing number of ADLs because of multiple, various, and evolving task needs, particularly in the frailty context [11]. Indeed, the restricted capability to perform ADL remains extremely patient-dependent (individual and need variability) [6]. Therefore, personalized multiple intervention programs are more efficient in reducing the impact of frailty progression (cognition, autonomy, and quality of life) than the usual intervention programs [12]. A third limitation related to the silo-based approach is context awareness of assistive services that are too restricted. Hence, such services are not flexible and are delivered irrespective of the actual person’s needs in a given situation, rendering them unsuitable or even obstructive for performing ADL [13]. Finally, informal and formal caregivers are important resources for community-dwelling FI, acting as *human support for ADLs* [14,15]. Thus, the assessment of ambient assisted living (AAL) benefits should integrate measures related to caregiver efficiency [16].

To advance the field of ATs, we designed a clinical trial assessing AAL-based multiservice assistance, called HomeAssist (HA), dedicated to supporting FI in their 3 main needs domains: ADL, safety, and social participation. We expected that HA use would result in better everyday functioning (main efficacy criterion: IADL scale score) compared with older adults living in the community not benefiting from HA. This paper describes the clinical trial design.

### Primary and Secondary Objectives

The objectives of the HA trial will be to perform a 12-month field study for testing in real conditions the use the HA-AAL in a sample of older persons (frail or not) living alone at home and then, to assess the impact and efficacy of HA in terms of aging in place and the efficiency of the caregiving environment.

Consequently, the primary outcome will be aging in place-related measures through functional status (IADL scale measure). Secondary criteria of HA efficacy will also be studied, including institutionalization and hospitalization as well as measures of health scales (self-efficacy, quality of life, routinization, etc), general cognitive status, memory, and executive functioning. In addition, caregiver-related measures will be used to assess the impact of HA on the daily delivery of services (eg*,* feeling of burden assessment and psychological health of caregivers) and reassurance regarding the situation of the older person.

Therefore, we mainly hypothesize that (1) the benefit of an AAL with a user-centered design is effective for aging in place of frail older adults in terms of everyday functioning (IADL scale score). As previously observed in pilot studies [17-20], we anticipate that the intervention group will maintain or improve their IADL scale score, whereas the control group will decline significantly. In addition, our secondary hypotheses are that (2) such an AAL decreases or neutralizes the frailty process and reduces the rates of institutionalization and hospitalization and that (3) it improves the psychosocial health of participants and their caregivers when compared with the control condition. Given these assumptions, we also assume that a large proportion of participants will want to continue the study at 24 months, despite the HA subscription fees.

## Methods

### Overview of the HA Study

#### Overview of the Functions of HA Services

HA is an implementation of general principles highlighted by an ecosystemic approach of human factors applied to the context of environmental gerontology that stresses environment-aging relations and encourages the synergy of multiple disciplines and professionals (eg, psychologists, epidemiologists, technologists, allied health professionals, community planners, and social policy makers). The common goal is the development of preventive and ameliorative interventions, targeting both individual and environmental factors to provide a better “fit” between FI and their home, thus supporting aging in place as well as good quality of life. Therefore, HA aims to provide services related primarily to promoting aging in place (independent living) and secondarily, improving the efficiency of the caregiving environment.

Accordingly, HA services support the autonomous realization of daily tasks, including social activities, which are known to be related to independent living capabilities and older adult well-being. Originally, applications support self-regulation and self-determination in helping users conform to their daily routines via sensor-based activity monitoring and assistive support (eg*,* activity reminders according to notification feedback from monitoring services customized according to user preferences).

The web-based HA catalog also offers applications materializing a caregiving proxy for several actions, including mutualizing the planning of care services, gathering information on older adult activities, reminding activities and appointments, and monitoring potentially unsafe activities and situations. The HA catalog and user interface designs have been primarily based on a human factor–centered approach to designing, introducing, and assessing an AAL platform among the FI.

#### Study Design

To test the impact and efficacy of HA, we designed a quasi-experimental study for a 12-month duration, which started in 2017, including older adults, ranging from autonomous to frail (ie, cognitive or physical frailty or both) equipped with HA, and matched controls recruited in Aquitaine territories (not equipped with HA), forming part of an existing population-based cohort on aging (The Three-City; 3C; study [21] and the aging multidisciplinary investigation [AMI] cohort [22]). Follow-up assessments for clinical outcomes (effectiveness on aging in place and caregiver burden measures) were performed at 0 (T0) and 12 months (T12).

In the HA condition, older adults and their families or formal caregivers had the option to participate in our proposed field study for an additional period of 12 months, only if they paid an HA subscription (corresponding to the cost of an internet subscription amounting to €20 or US $ 20 a month). This “paid” aspect of the study design allows for evaluation of the perceived usefulness of HA by the participants leading to its purchase and long-term adoption. Hence, follow-up assessments for equipped participants occurred at 0 and 12 months and optionally at 24 months. Follow-up assessments for user experience, attitudes, and HA use were also collected every 6 months (0, 6, and 12 months and optionally at 18 and 24 months).

#### Eligibility Criteria

First, the participants had to be aged ≥65 years of age or older; live alone in an independent community setting (with an equal selection in urban, semiurban, and rural locations); have a Mini-Mental State Evaluation (MMSE) score >23 [23]; be able to understand the use of applications and devices included in the HA platform; be ambulatory (either without support or with a cane or a walker); have an available, reliable formal and informal caregiver (contact frequency>4-5 hours per week); and be able to understand and sign informed consent.

In addition, they had to have prefrailty or frailty syndrome according to the Short Emergency Geriatric Assessment scale [24].

The exclusion criteria were as follows: living in an institution (nursing home), living with a partner, having an upcoming relocation project, having from dementia, having visual or hearing loss or any other conditions limiting HA use, or having ongoing serious or unstable medical conditions or any personal condition that may limit follow-up visits.

#### Constitution of the HA and Control Groups

For HA conditions, several recruitment methods have been used, such as advertisements in local media, interactions with home services for aging in place, and attendance at older adult associations. Analysis of the recruitment data indicated that home services specialized for older adults were the most fruitful recruitment process. These home services were equally located in rural, semirural, and urban areas. The recruitment duration was 12 months.

The best way to assess the efficacy of an AAL is to compare the individual evolution while being equipped with an AAL compared with nonequipped persons. To do so, a control group has been constituted from 2 existing epidemiological population-based cohorts on aging: the 3C study [21] and the AMI cohort [22]. Briefly, 9294 participants of the 3C study, aged ≥65, initially noninstitutionalized, were selected from the electoral rolls in 3 French cities (Bordeaux, Dijon, and Montpellier; N=2104) and included from 1999 to 2000. The AMI cohort included 1002 retired farmers, aged ≥65 years, randomly selected from the Farmer Health Insurance System and followed up since 2007. In each cohort, several follow-up examinations were performed every 2 to 3 years with visits conducted at home by trained psychologists.

The control group of this trial has been constituted from the sample of 3C participants living in the Bordeaux site, not equipped with the HA solution, and interviewed at both the 10-year and T12 follow-ups and from the AMI sample, using the baseline and T12 follow-ups. Following the HA eligibility criteria, the control group included ≥70 older persons living alone in an independent community setting. Comparisons between the 2 groups (intervention vs control) are possible because of similar assessment tools between studies regarding the primary and secondary outcomes, for IADL scale, cognitive performance, depressive symptomology, hospitalization, and institutionalization.

### Description of the HA Intervention

#### The HA Platform

HA services are apps developed using the DiaSuiteBox technology [25], except for some services for social participation where existing apps are used (see further sections). HA is an AAL platform based on pervasive computing leveraged from the Internet-of-Things (IoT). As a result of previous user-centered design studies [18,26-33], HA proposes many assistive apps in the 3 key domains of everyday life, as follows:

- *Daily activities*, including circadian activity monitoring (getting up from or going to bed according to sleep habits declared by the user and toilet activity), daily routine monitoring (3 meal preparations: breakfast, lunch, and dinner; getting dressed; and bathing or showering) with the option of asking the activity reminder to issue a notification when the activity is not performed in the desired time slot by the user [29,30], appointment, and event reminders using a simplified diary app, shared with the informal caregiver. Indeed, a simplified diary application has been specially developed so that both family caregivers and techno-clinicians can quickly and easily enter the activities and events and their recurrence to be recalled.
- *Home or personal safety*, including entrance monitoring (entrance door left open, daytime outings, or abnormal night outings), electric appliance alerts (fridge, stove, or coffee maker monitoring), a light path for night getting-ups, and no activity alert to the caregiver when the user does not answer.
- *Social participation*, included internet browsers, photo sharing with family, collaborative games (cards games, Scrabble, arrows and crosswords, puzzles, etc), videoconferencing (Skype), simplified mailing systems (eg, voice recording to send messages and speech synthesizers to read the messages out loud [28], simplified email information about local events, television programs, bank service, etc) [18].

**Figure 1:**
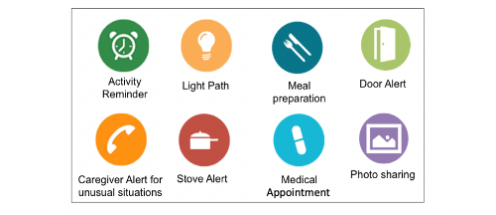
Examples of assistive applications from our web-based catalog.

Assisted by a specifically trained techno-clinician, older adults and their caregivers were asked to determine what and how activities should be assisted by selecting the appropriate assistive applications and configuring them with respect to the person’s needs and preferences. The resulting set of applications provides personalized assistive support for an individual while respecting the self-determination of the older person [18]. In addition, to respond to evolving needs, our platform allows to stop or remove applications easily and to install new ones from the web-based catalog. Figure 1 represents a more typical app pack chosen by older adults and their caregivers.

It is noteworthy that both the activity and the daily routine monitoring services are systematically deployed in each home, as they are at the heart of the HA system, while the assistive apps and caregiver alerts can be optionally selected for personalized assistance. Indeed, the situational awareness provided by a monitoring service (ie, passive user interactions) is a key property of HA for ensuring relevant assistive services by Belloum et al [33]: (1) answering queries such as “is the person asleep?” (2) recognizing ADLs; (3) automatically detecting alerting situations such as door opening during the night; and (4) identifying specific behavior changes that may indicate, for instance, a decline in health status such as changes in ADL routine or a reduction in the number of times going out of the home [29,30]. Abnormal behaviors are detected according to the classification by Tran et al [34], who have defined four types of abnormal behaviors: (1) known behavior in a deviating spatial context (eg, sleeping in the living room), (2) known behavior occurring at a deviating moment in time (eg, leaving home at an abnormal time or having dinner unusually late), (3) known behavior with an abnormal duration or occurrence (eg, sleeping until noon or going to the toilet twice as many times as before), and (4) behavior resulting in abnormal or unexpected sensor firing patterns (eg, a fall resulting in an extended period of mute sensors).

The minimal HA setup included at least a daily routine monitoring service with no activity reminder notification, 1 to 2 security applications (most often, door alerts and no daytime activity alerts), 2 to 3 social activity applications (simplified mailer, internet browser, and games), and photo sharing on the main tablet. Table 1 summarizes the main HA and its commercial apps deployed or potentially deployed in homes.

**Table 1:** Summary of apps included in the HomeAssist (HA) platform assistive technologies (ATs).

Tablet support and activity domain				Apps developed into the HA platform		Features				
						Home service		Installation		Type
**Main tablet (I)**										
	**Daily activity**									
		Circadian activity	Getting up from or going to bed		Monitoring		✓		HA	
		Circadian activity	Reminder		AT		Optional		HA	
		Continence activity	Toilet activity		Monitoring		Optional		HA	
		Continence activity	Reminder		AT		Optional		HA	
		Dressing activity	Clothing furniture’s activities		Monitoring		Optional		HA	
		Dressing activity	Reminder		AT		Optional		HA	
		Bathing or showering activity	Bathroom activity		Monitoring		Optional		HA	
		Bathing or showering activity	Reminder		AT		Optional		HA	
		Meal preparations	breakfast, lunch, and dinner		Monitoring		Optional		HA	
		Meal preparation	Reminder		AT		Optional		HA	
		Daily routine	Three meal preparations and circadian activity		Monitoring		✓		HA	
		Daily routine	Report		AT		✓		HA	
		Simplified diary	Appointment and event reminders		AT		✓		HA	
	**Safety**									
		Entrance door	Door left open, daytime outings, or abnormal night outings		Monitoring		✓		HA	
		Entrance door	Reminder or alert		AT		✓		HA	
		Electric appliances	Fridge, stove, or coffee maker (end user: frail individual)		Monitoring		✓		HA	
		Appliance	Reminder or alert		AT		✓		HA	
		Light path	Night getting-ups		AT		Optional		HA	
		No-activity alert	No daytime activity from any sensor (end user: frail individual or also caregiver)		AT		✓		HA	
	**Social participation**									
		Photo sharing	With family or automatic pushing according to person’s interests		AT		✓		HA	
		Email notification	New received email		AT		✓		HA	
**Secondary tablet (II): social participation**										
	Internet		Internet browser (Google)		AT		Optional		Commercial app	
	Asynchronous communication		Simplified email (relatives and local public services for leisure and social activities)		AT		✓		HA	
	Synchronous communication		Video conferencing (Skype) and social network (Facebook)		AT		Optional		Commercial app	
	Financial or administrative activities		Bank and government apps (regular administrative procedures, incomes taxes, etc); collaborative and noncollaborative games (cards games, Scrabble, arrows and crosswords, puzzles, etc); video player (YouTube); television programs		AT		Optional		Commercial app	

#### Sensors and Actuators

**Figure 2:**
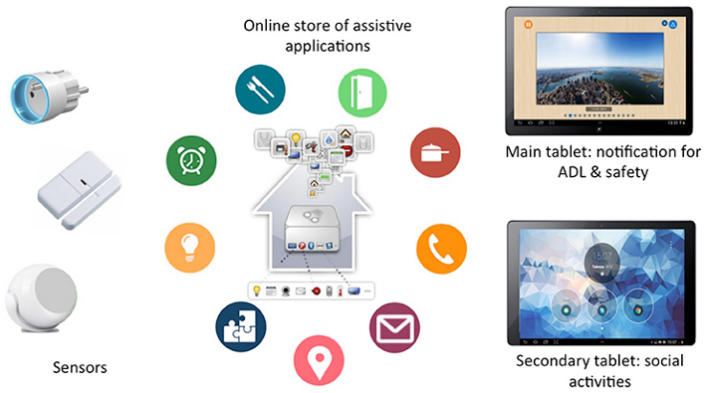
Content of HomeAssist platform—a set of sensors, a web-based catalog of assistive applications, and two touch screen tablets (main and secondary). ADL: activities of daily life.

Technically, monitoring and assistive applications rely on the infrastructure of the devices and web services deployed at the home of each user. The HA platform relies on a set of sensors and actuators, as well as 2 touch screen tablets (Figure 2). Indeed, several entities are required for running HA services: (1) wireless sensors (motion and contact sensors and smart electric switches) and 2 tablets and (2) software services (agenda, address book, mail agent, and photo agent). Three types of sensors were used in our platform: contact sensors that enable detection when a door or drawer is opened or closed, electric meters that sense the electric consumption of electric appliances and enable them to remotely turn it on or off, and motion sensors that collect timed information when motion is detected in their sensing range. These sensors were chosen because they are small, wireless, and cheap, and they respect users’ privacy.

**Figure 3:**
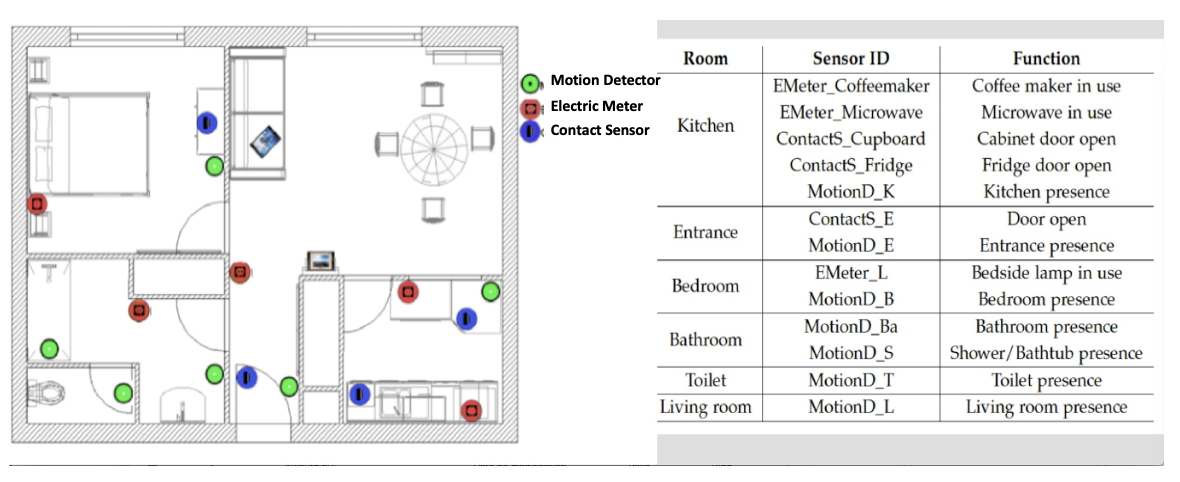
Typical HomeAssist (HA) kit deployed, with apartment layout with sensors (left) and the HA sensors and their locations and functions (right).

Sensors are placed in strategic locations: kitchen, bedroom, bathroom, toilet, and near the entrance. Figure 3 shows a typical HA kit that is deployed.

#### Two Touch Screen Tablets

Regarding active user interactions, users interacted with the platform via 2 similar touch screen tablets operating under Android OS, which we configured following the guidelines for older population (eg, International Standardization Organization/TR 22,411 [35]; Accessibility World Wide Web Consortium [36,37]) and prior user testing relative to objective and subjective measures of usability, use learning, and self-reported user experience [18,28,31,32]. Importantly, HA services revolve around a unifying and simplified interface suitable for older adults [31]. Having 2 tablets allows the person to associate each tablet with a type of service (assistive apps for daily activities and safety on the main tablet vs social activity apps on the secondary tablet). In addition, this approach also brings two levels of security: (1) assistive services guarantee for users—the main tablet remains stationary and always under electric power ensuring the nominal delivery of the notifications, while the secondary tablet is used freely in the space, and if it turns off in case of empty batteries, it does not have critical consequences for the person and (2) HA service reliability—all the apps on the main tablet are specially developed into the HA platform, where we controlled the security process of the data collected while the secondary tablet included commercial apps for which it is not possible to have a fully secure control.

**Figure 4:**
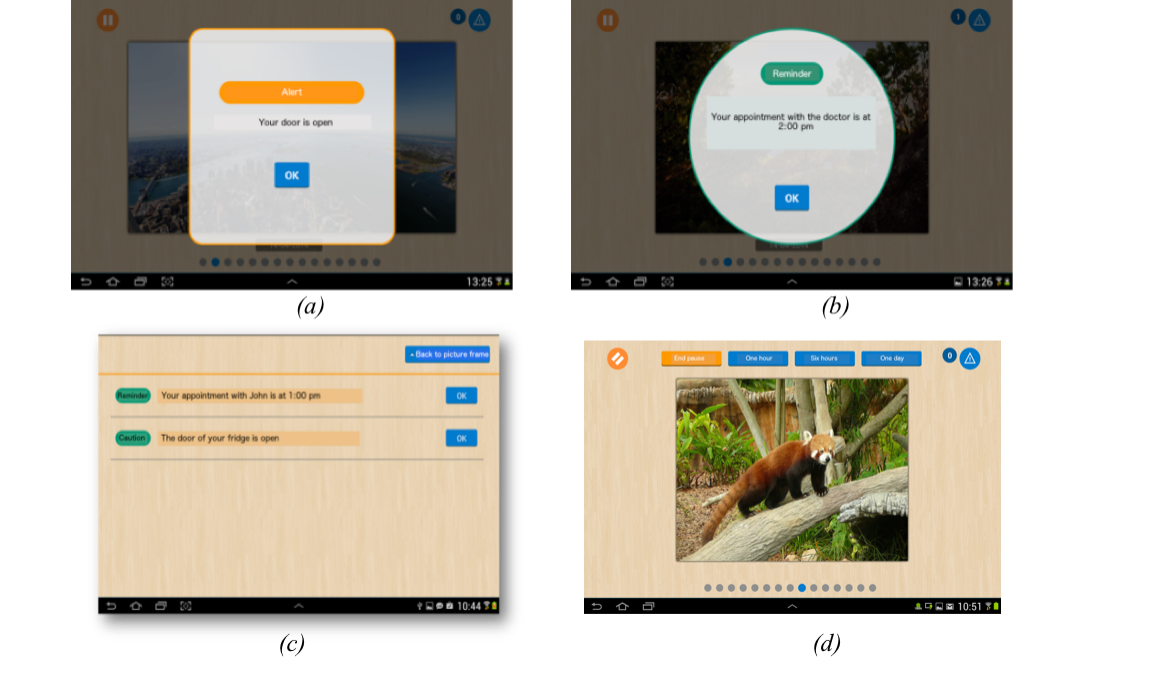
Critical notification (A), noncritical notification (B), list of unattended notifications (C), and pause feature of the notification system (D).

For the main tablet where the assistance notifications are displayed, the notification system exploits the preference of older adults for simple interactions and optimizes their cognitive resources using multimodal coding of notifications (tones, shapes, colors, and text). All assistive applications are required to interact with the user via either a critical or noncritical notification, depending on the risk level of the situation. Each type of notification uses a specific multimodal coding, as illustrated in Figures 4A and 4B, displaying a critical and noncritical notification, respectively. This approach makes it easier to discriminate among the notification types. Furthermore, the user follows a dedicated procedure for each notification type. Critical notifications (Figure 4A) use a loud volume and only disappear when the situation is resolved; they can contact a caregiver via an SMS text message after a predefined period to seek help. By contrast, noncritical notifications (Figure 4B) use a soft tone; they disappear after being displayed for a set period and are added to a list of unattended (noncritical) notifications. An example of such a list is shown in Figure 4C. This mechanism allows the user to disregard a notification if it occurs while performing another task. If the condition that raises a noncritical notification does not hold (eg, the door of the fridge is closed), then this notification is suppressed from the list of unattended notifications. To respect user self-determination [18], the notification system can be deactivated by the users themselves for a predefined period, for example, when someone visits the user, as depicted in Figure 4D. When no notification is delivered, the main tablet turns into a photo display shared with the person’s relatives, allowing discreet and nonstigmatizing assistance.

**Figure 5:**
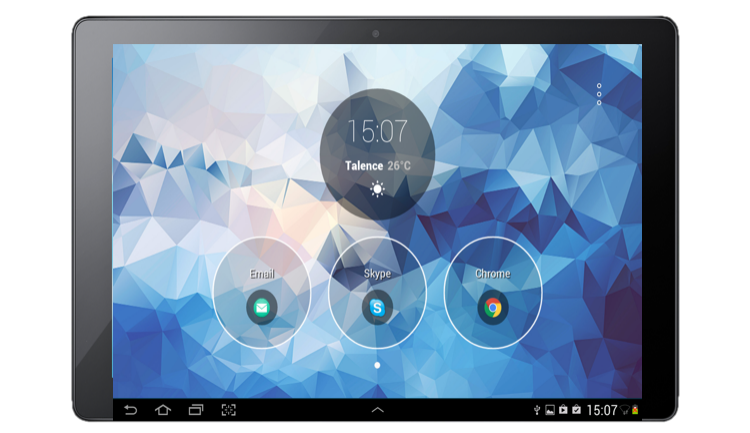
Secondary tablet with its simplified app launcher.

The secondary tablet provides social participation services (except for the photo-sharing app). A simplified application launcher was developed to make it easier to use these services (Figure 5). This launcher displays applications as a page listing 3 applications. A simple click on the icon of the application opens it. A total of 5 pages can be created, and the user navigates from page to page with a simple gesture of the finger, mimicking leafing through a book. The launcher updates itself as it is used and lists the applications on the pages according to their use frequency so that the user finds the applications that are used most often more quickly.

A video presentation of the HA is available to provide a concrete idea of the HA platform [38].

#### Protocol and Time Line Procedure

For each participant, several visits were planned: 1 for eligibility, 2 for personalized installation of HA services, 4 to 6 for training, and 1 to 2 for each follow-up.

##### Eligibility and Baseline Assessment

Eligibility criteria were screened in 2 steps. First, the participants who were interested in participating contacted the study coordinator, who screened the first eligibility criteria (age, independent home, living arrangements, and geographic area of residence). Then, an eligibility visit was conducted at the participant’s home with a techno-clinician to apply all the eligibility criteria (cognitive and functional status, frailty level, etc) and the procedure for obtaining informed consent. For this eligibility visit, the person was advised to be accompanied by a person of trust or by a close person who frequently assists them (family, neighbor, friend, etc). The presence of a relative had a 3-fold objective: to reassure the older person; to help the older person, if necessary, to make their decision to participate in the study; and finally, to invite this relative to participate. A period of 7 days of reflection was given to each pair of participants to formulate their own decisions. When the eligibility visit was successful, the other visits were scheduled.

##### HA Service Selection

First, the techno-clinician carried out the steps to subscribe to the internet for each participant. The costs of this subscription were covered by the research program, and the participant had nothing to pay for 12 months, but its renewal for another 12 months was at the participant’s expense if they wanted to continue the experiment.

The clinician’s goal was to make explicit and record older adults’ everyday routines, desired assistive services, and preferences, notably in terms of both critical and noncritical notifications and notification sharing with a caregiver. The presence of a caregiver (family, friend, or home care professional) was greatly advised, both to reduce older adult’s stress and to help the older adult declare their preferences.

The choice of assistive services was based on a needs questionnaire, in which each need was associated with a pictorial description of the corresponding HA service. The questionnaire was divided into 2 parts.

The first part assessed the needs in terms of daily routine, everyday activities, and safety, for which assistive services are provided on the main tablet.

**Figure 6:**
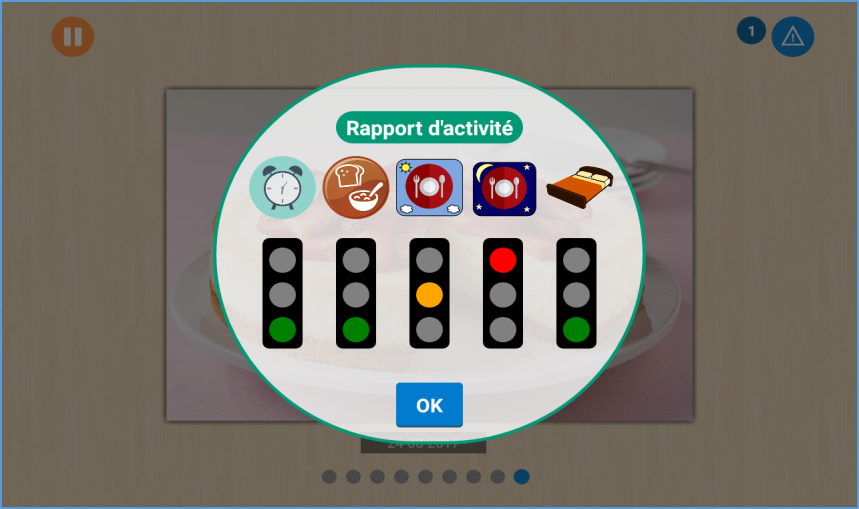
Example of a daily routine report provided to the user on the main tablet.

For daily routine, 4 types of activities were systematically monitored (getting up and going to bed, the 3 meal activities, bathing activities, and dressing activities), the performance of which was monitored every day, and an assessment was reported to the person in a pictorial way, where each activity was evaluated in the form of traffic lights (green: 80% of the activity has been performed; orange: 50%-79% of the activity has been performed; red: <50% of the expected activity has been performed; Figure 6). Each participant can then choose to have a reminder of each of their routine activities on the main tablet and send the daily activity report to the caregiver of their choice.

Moreover, reminder services (simplified diary application) were dedicated to medication intake or reminders of any activities (medical appointments, hairdresser appointments, leisure activities, etc) or events (relatives’ birthdays, a television show, local social events, etc).

For safety, several notification services are offered: monitoring household appliances, the unintentional opening of the front door or fridge, abnormal absence of activities in the home during the day, and repeated nighttime getting-ups. In addition, a service for activating a light path during nighttime rise is offered. For each notification or reminder-based service, the participants decided whether the notification was critical. If the notification had a critical status, the participant could choose whether the notification was sent via SMS text message to the phone of their informal caregiver. The first part of the questionnaire ended with the participant’s choice of photos displayed on the main tablet and the schedule of their updates. To do this, the participant indicated which relatives could send photos, and if no relatives were present, the participant indicated their interests, from which photos were extracted from free photo libraries.

The second part of the questionnaire referred to services dedicated to social participation activities provided by the secondary tablets. These services include internet browsers, communication applications (videoconferencing or a simplified mailer), and various applications such as television program guides, simple games (card games, puzzles, crossword puzzles, etc) and interactive ones (scrabble, card games, etc), shopping services (food, clothing, etc), and banking or public services applications (income tax, social assistance, etc). Overall, each HA service setup was personalized. The minimal setup included at least a daily routine monitoring service with no activity reminder notification, 1 to 2 security applications (most often, door alerts and no daytime activity alerts), 2 to 3 social activity applications (simplified mailer, internet browser, and games), and photo sharing on the main tablet.

The questionnaire-based service selection could be updated every 6 weeks during the home visit by the techno-clinician.

##### HA Installation

When the services were chosen, the second home visit was organized with a home automation technician to install the HA platform in the participant’s home and test whether the platform was working properly. Each participant’s home in the HA condition was equipped with an HA kit, including sensors, actuators, and 2 tablets, as described previously. All participants also had a stylus available to interact with the tablets as needed (according to our pilot study, some very old participants had fingertips that did not allow effective interaction with the tablet). From a technical point of view, the necessary steps included setting and personalizing the assistive apps (eg, filling the calendar or adding family contacts) and testing the platform. Simultaneously, the techno-clinician initiated the training phase.

#### Training and Practice of HA Services

Again, the presence of a caregiver was advised to learn from the techno-clinician and later to offer support on technical issues. The training phase for using the HA services was gradual. During the training phase, the techno-clinician introduced the different features of the platform on a step-by-step basis during 4 monthly sessions of 75 minutes based on concrete scenario use. This instructional strategy was successfully used in other studies (eg, [38]). The use of services offered by the main tablet (daily activities and safety-related services) was first targeted. Essentially, simulations of assistance scenarios lasting 10 minutes placed the participant in a situation of active interaction with the notification (critical vs noncritical) and pause features of HA [18,29]. The techno-clinician was provided with a scale to evaluate the quality of the response (time taken and number of errors, omissions, and commissions), and if this was unsatisfactory, the simulations were repeated. A maximum of 3 repetitions was sufficient to obtain and understand the appropriate interactions from the participants. The techno-clinician left a pictorial user manual of the services, especially designed for the participants, on which a dedicated space allowed the participant to write comments and difficulties encountered with the services provided by the main tablet. During the first training phase, only the main tablet support services chosen by the participants were active. A week later, a third visit was arranged by the techno-clinician who checked the user manual to see if any difficulties had been encountered with HA. If so, the first phase of training was repeated. If this was not the case, the second phase of training began, which focused on the use of social participation services on a secondary tablet. Here again, scenario simulations were proposed in the spirit of the first training phase, aiming at the proper use of the application launcher, the opening of an application, and its use. This second phase lasted longer, 20 to 30 minutes, because for the email and videoconference applications, the techno-clinician entered the person’s desired contacts and carried out numerous tests with them. For services from the main tablet, a picture-based user manual for the secondary tablet was provided to each participant (a usability expert initially designed both user manuals). At present, all the social participation services on the secondary tablet were active. At the fourth visit, the techno-clinician checked to see if the person had difficulty with the secondary tablet. As the interfaces of the selected applications (especially the applications not designed by us, eg, game) had very heterogeneous designs; often the second training phase had to be replayed during this fourth visit. When this was not the case, the third phase of training was initiated. This phase always consisted of simulated use scenarios but mixed the services of the main tablet with those of the secondary tablet. If the participant’s interactions with the platform were appropriate, the training ended. Otherwise, the fifth visit was scheduled. Hence, HA training took a minimum of 4 sessions (1 per week) and a maximum of 6 sessions.

#### A Support Hotline

Throughout the training and study duration, a 24/7 support hotline was available to all participants. Thus, problems were solved remotely, but some required personal visits for issues such as changing sensor batteries, the loss of internet connection, and the participants’ incorrect use of the tablet, leading to malfunctions. In all cases, participants received a “check-in” call at 1 week, 1 month, 6 months, 12 months, 18 months, and 24 months.

### Assessment of the HA Intervention

**Figure 7:**
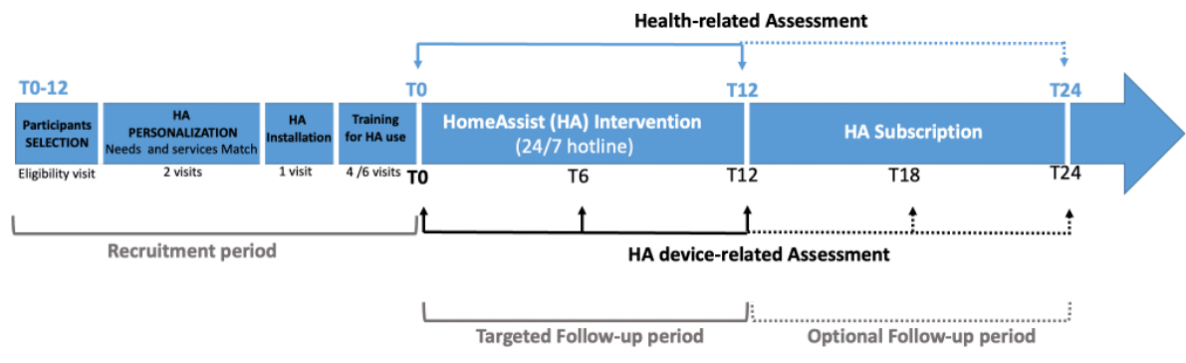
Time line of recruitment and follow-up for the HomeAssist (HA) condition.

The assessment is divided into 2 parts: health (Multimedia Appendix 1 [22,38-59]) and HA device (Multimedia Appendix 2 [17,18,60-73]) parts (Figure 7).

#### Health-Related Part of the Assessment

Initially, the participants completed a *screening questionnaire* that assessed their basic demographic information and living home conditions. These measures will serve as potential moderating variables in our analyses.

They also completed the efficacy follow-up battery, including independent living capabilities, frailty-related ratings, and self-perceived health for the participant and the informal caregiver. The primary outcome measures for the trial include changes at 12 months (and optionally at 24 months) in everyday functioning (self-perceived and perceived by informal caregivers measured using the IADL scale [39]). The other measures were the secondary outcomes.

This health-related assessment is already part of the 3C and AMI cohorts, from which the control group is formed.

#### HA Device Part of the Assessment

An additional set of measures is provided to the participants equipped with the HA system. This additional assessment aims to study across time (at 0, 6, 12 months, and optionally at 18 and 24 months) the user’s HA needs, the user’s HA perception, the HA uses and usages, and their possible relationships and the evolution over time. HA uses and usages are assessed through active interactions with the HA platform to which passive interactions are added, particularly the monitoring of activity in the home via motion sensors or door or drawer contactors. These latter measures are called HA-related measures.

### Treatment Fidelity and Data Collection

For the HA condition, an external trial monitoring board has been specifically set up and met once a year or as needed. It included 12 members gathering multiple expertise (gerontology and geriatrics, clinical trials for behavioral interventions, home services, public services for older adults, user-centered gerontechnology, IoT and software orchestration, epidemiology and statistics, and innovation transfer). This board provided trial oversight and monitored participant safety and well-being for the entire trial duration.

A detailed manual of operations has been developed for all study protocols, and HA-related implementation and training protocols have been scripted. All study activities are discussed at weekly HA coordinating team meetings with the project coordinators, the data management team, and technical staff around issues related to data collection, transfer, or HA technology.

### Statistical Treatments

Descriptive analyses were conducted to characterize the recruited HA sample compared with the others (excluded and refusals) on the data available at the eligibility visit and to describe the characteristics of the recruited sample at baseline as well as those of the dropouts. We also performed group comparisons to obtain a baseline description of the HA and control groups for the primary and secondary outcomes.

To test our main and secondary hypotheses, all measures of the study will be tested based on an intention-to-treat approach using a 2-tailed level of significance set at α=.05. The quasi-experimental study design will allow the comparison of primary and secondary efficacy outcomes in HA and control conditions. The 12-month follow-up data being not available for the control sample, imputations will be performed according to a linear hypothesis of the evolution of the scores between T0 and T24. For each outcome, we will compare the mean score at T12 between the HA and control groups. In addition, to control for confounders, three successive linear regression models will be used: (1) Model 1: Y_(at T12)_ = DomAssist (vs control) + age + Sex, (2) Model 2: Y_(at T12)_ = Model 1 + dependent variable at T0 (Y_(à T0)_), and (3) Model 3: Y_(at T12)_ = Model 2 + MMSE T0.

Finally, we will also describe the evolution of the primary and secondary efficacy outcomes over 12 months of HA use and conduct comparisons by age, gender, initial level of technology acceptance, and user satisfaction with HA technology (collected after 6 months of use).

### Ethics Approval

For the HA condition, the French Southwest and Overseas Protection of Persons Committee deemed the study to be outside the scope of the provisions governing biomedical research and routine care (DC 2015/2066). The study protocol was also approved by the National Commission of Informatics and Liberty (Comission Nationale Informatique & Libertés-French name) and the Ethics COERLE (Comité Operationel d’Evaluation des Risques Légaux et Ethiques) committee of the French National Institute of Informatics and Mathematics (Inria), as protecting participants and data accordingly. For the control group from the 2 cohorts, an ethics committee approved the research according to the principles embodied in the Declaration of Helsinki: for 3C, the Ethical Committee of the University Hospital of Kremlin-Bicêtre (Paris, France) and Sud-Méditerranée 3 (Nîmes, France) and for AMI, the committee of the University Hospital of Bordeaux (France). All the participants provided written informed consent.

## Results

The HA project is funded for a 5-year period (2016-2021), and the full HA intervention (24 months) was completed in 2020 before the COVID-19 crisis.

### Participants Recruitment

**Figure 8:**
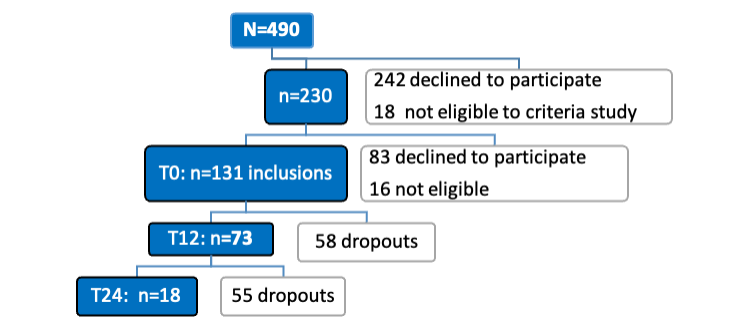
Recruitment diagram for HomeAssist condition.

As indicated in Figure 5, a total of 490 individuals received a prescreening visit for the HA condition. Of these 490 individuals, 260 (53%) were excluded because of ineligibility (n=18) or a lack of interest in participating (n=242). A total of 230 older adults underwent the baseline assessment, of which 99 (43%) were excluded (16 ineligible; 83 refusals). Finally, 56.9% (131/230) of individuals were enrolled: more than half of the participants completed the 12-month follow-up (73/131, 55.7%), from whom one-third of participants (24/73, 33%) were requested to continue the experiment for 6 additional months (18 months), and 18 participants were enrolled for 12 additional months (24 months). Figure 8 illustrates the recruitment flowchart for the 24-month duration of the HA condition.

The HA sample (n=131) at T0 is primarily female (114/131, 87%) and ranges in age from 70 to 93 (mean 81.9, SD 6.0) years. The participants enrolled in the HA condition have varied living spaces: 35.1% (46/131) individuals in rural areas, 27.4% (36/131) in semirural areas, and 37.4% (49/131) in urban areas. Of the 131 participants, 63 (48.1%) do not have a senior high school degree, and 57 (43.5%) do not have a junior high school degree. The MMSE scores range from 23 to 30 (mean 25.9, SD 2.4).

Of the 131 participants, 58 (44.3%) dropped out before T12. Compared with T12 completers (73/131, 55.7%), these individuals were slightly older (26/58, 47% were over 85 years vs 16/58, 22% of T12 completers; *P*=.004) and less educated (mean 8.2, SD 3.2 vs mean 9.8, SD 2.3; *P*=.005), while no significant difference was observed in gender (*P*=.80), living environment (*P*=.26), frailty-Short Emergency Geriatric Assessment score (*P*=.10), and MMSE score (*P*=.18).

### Baseline Description of HA and Control Group

**Figure 9:**
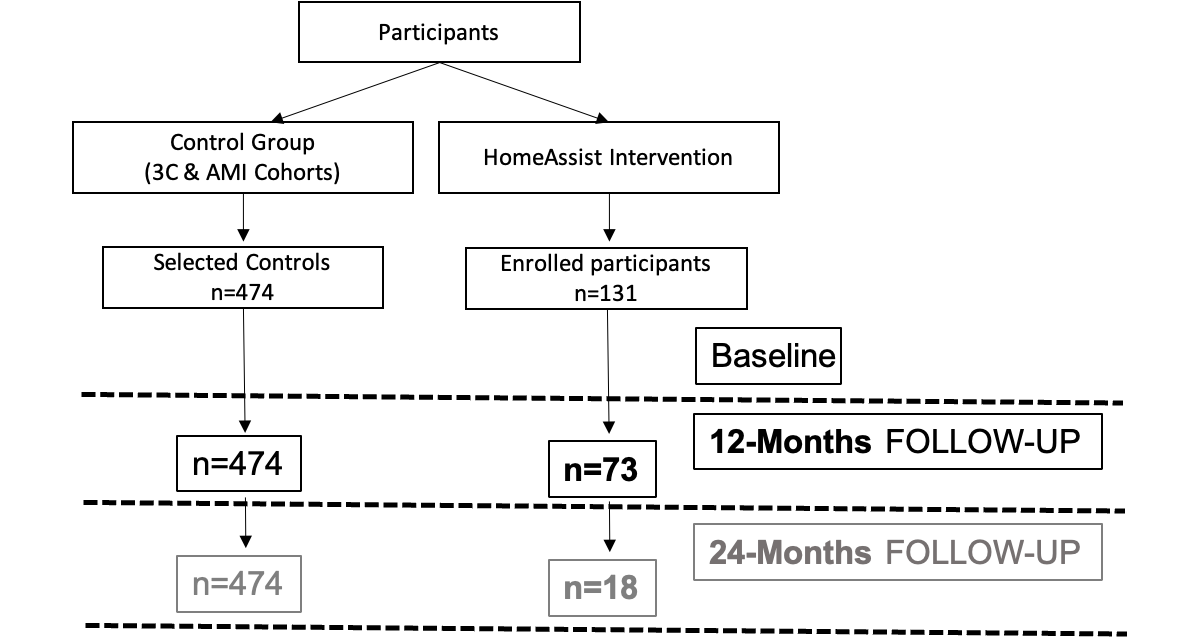
Flow of the study. 3C: Three-City; AMI: aging multidisciplinary investigation.

From the 3C and AMI cohorts, 474 control participants were selected according to the eligibility criteria (Figure 9).

When comparing baseline characteristics between the HA sample and the control group, we observed no differences in terms of age, education, living condition (all living alone), IADL disability, depressive symptoms, and cognition. For the MMSE test, the control group tended to have a higher mean score, but this difference was not significant (*P*=.08). However, as indicated in Table 2, participants in the HA condition were more often women (63/75, 88% vs 372/474, 78.4%; *P*=.054).

Regarding our 2 primary outcomes, the sample size provides a reliable statistical power of 0.80 with a significance level set at .05, attesting to a significant difference in means of the IADL scale score between the 2 groups for a difference >0.87 units.

**Table 2:** Sample description and group comparisons for baseline measures of primary and secondary outcomes.

	HA^a^ (n=73)		Control (n=474)		*P* value
	Sample size (n)	Value	Sample size (n)	Value	
Age (years), mean (SD)	73	81.2 (5.1)	474	81.3 (4.3)	.84
Age (≥80 years), n (%)	73	43 (58.9)	474	303 (63.4)	.46
Sex (female), n (%)	73	64 (87.7)	474	372 (77.8)	.05
Lower level of education, n (%)	62	27 (43.5)	474	229 (48.3)	.48
Living alone, n (%)	73	73 (100)	474	474 (100)	—^b^
IADL^c^ disability, n (%)	67	21 (31.3)	473	137 (29)	.68
IADL score, mean (SD)	64	6.4 (2.2)	473	6.4 (2.5)	.92
CESD^d^ score, mean (SD)	73	10.0 (6.7)	427	9.1 (8.9)	.38
MMSE^e^ score, mean (SD)	66	26.0 (2.4)	474	26.6 (2.6)	.08
Benton score, mean (SD)^f^	68	10.8 (2.2)	318	11.2 (2.3)	.12
Isaac^g^ 15-s score, mean (SD)	72	26.3 (6.1)	459	26.2 (6.4)	.94
Isaac 30-s score, mean (SD)	72	40.6 (8.5)	459	40.1 (10.4)	.68

^a^HA: HomeAssist.

^b^Not available.

^c^IADL: instrumental activities of daily life.

^d^CESD: Center for Epidemiologic Studies—Depression Scale [41].

^e^MMSE: Mini-Mental State Evaluation.

^f^Benton test [40] was not available for the aging multidisciplinary investigation cohort.

^g^Isaac fluency test [74] (see Multimedia Appendix 1 for details).

## Discussion

### Principal Findings

The HA project proposes an ecosystemic human-centered approach for introducing a personalized AAL platform dedicated to FI and their caregivers. Hence, our main outcome is to observe the benefit of our HA approach on this capability (improvement or nondeterioration) due to IADL follow-ups performed at 0 and 12 months. Our secondary expectation is to observe a positive effect of HA intervention for prolonging aging in place of FI living alone through improved cognitive and mental health, leading to reduced institutionalization and hospitalization. We also anticipate a positive effect on caregivers’ health. In addition, owing to the personalization of HA services and their user-centered design, we also expect that equipped persons will have a positive user experience on pragmatic criteria (perceived usability, usefulness, ease of use, learnability, etc) and hedonic criteria (pleasure, satisfaction, perceived attractiveness, etc). These expectations are directly based on our previous pilot study involving 17 HA-equipped FI compared with 17 nonequipped FI to assess the feasibility of the HA concept and collect preliminary results regarding its acceptance and elicited user experience. The results showed that HA was well adopted (highly accepted and usable) by FI and their families or caregivers [18]. Moreover, after 6 and 9 months of follow-up, the benefits of measures of self-determination behaviors and IADL scale scores [9,19] were higher in equipped FI [18-20]. For instance, with normalized scores, at 9 months, the equipped group did not change its IADL scale score, whereas the control group lost 1 SD. In addition, the objective burden of caregivers to support care receivers’ ADL increased in the control group after the follow-up period, while it did not increase in the equipped group, even though the subjective burden remained unchanged [19,20].

### Comparison With Previous Work

A total of 131 users and their families or formal caregivers participated in our proposed field study, and 73 accepted to continue the experience until 12 months. Such a clinical validation study is rare in the field of AAL-based interventions in terms of experimental design quality. Indeed, most studies in the AAL field remain at the prototype level (because of technological and usability or acceptability challenges) or at best pilot studies (because of the challenges of a field study) with small samples that do not provide the onset of the ground truth of AAL-based interventions [75].

Technological barriers include issues related to the deployment of the technologies, such as inaccurate sensors; battery power or bandwidth reliability issues, restricting users within the monitoring area; and lack of interoperability [76]. The lack of user-centered design approaches in AAL development also contributes to the low usability observed in some instances because of very limited experience for older adults in using advanced technologies [77,78], and even more for FI with physical or cognitive exhaustion leading to a lack of motivation to accept actively interacting with such technologies [79]. Taken together, these barriers have led field studies with large sample sizes to adopt a silo approach offering specialized services in one domain of need for older adults. For instance, the Personal Reminder Information and Social Management system only provides services for communication and leisure [80], and the platforms designed by Rantz et al [81] and Tomita et al [17] focus on activity monitoring. In addition, the efficacy of such specialized AAL is always estimated by a focus on older adults, without involving an impact evaluation of caregivers’ well-being. However, as highlighted by Fadrique et al [76], AAL benefits must be measured for the social care environment while protecting (near and far stakeholders) against risks in privacy and security related to data sharing in the AAL scope.

Therefore, multidomain AAL is still only in the design phase, as recently proposed with the next system study protocol [82].

### Strengths and Limitations

The HA platform has already completed all the design, user testing, and pilot study phases to reach the empirical evidence step based on this study. Furthermore, our field study includes evaluations that cover other critical indicators for home care more broadly, such as the rate of institutionalization in nursing homes, the impact on mental and physical health, and data reported by HA (actimetrics, use, and use of services). For the equipped group, we gathered data on the factors that influence usability, technology acceptance, and use. These multiple secondary indicators aim to respond to the needs expressed by all the project’s stakeholders (aging policy makers, home service organizations, caregivers, older adults, and researchers from different domains) for successful participatory achievements while also moving on to more daring, innovative plans; for example, new research areas such as the study of relationships between AAL acceptance and AAL effectiveness for aging in place. In addition, the field and participatory nature of this study imply combining a traditional top-down approach (ie, expectation relative to specific primary criteria for a prescriptive purpose) with a bottom-up approach definitively driven by the field expertise of the main stakeholders of aging in place. The growth of citizen science supports that this combined approach (albeit risky) is a successful way to bridge science and practices with the result of rapid societal impact for targeted people and for emerging new research issues [83].

From our combined approaches, the study consortium encountered a few issues that reinforced study-related challenges, especially with older adults and with technology-based intervention. First, major challenges are related to the quasi-experimental study design, where the HA condition is compared with the control condition of a subsample of an existing population-based cohort on aging. We paid great attention to matching as well as possible the 2 groups with respect of the factors known to influence the IADL scale score (main criteria outcome). These factors include demographic factors, frailty scores, and cognitive status*.* Nonetheless, we are aware that such a matching method does not neutralize possible biases related to differences in recruitment methods, the number of home visits on 1-year deployment, or the minimal level of technology acceptance that is probably shared by the participants in the HA condition. It should be remembered that out of almost 500 older adults contacted, only 46.9% (230/490) met our selection criteria, and only 26.7% (131/490) agreed to participate in the study. Half of the participants dropped out after 12 months of experimentation, notably regarding technology-related challenges.

A second challenge is a great variability in participants’ technological skills in the HA condition. As recommended [84,85], we set up a standardized training phase for the use of HA, and each participant was guaranteed to keep the 2 tablets provided for the study after the experiment to boost their interest in such technology training. In addition, the interoperability of HA with a variety of technologies (Bluetooth, UPnP, ZWave, Web services, etc) and its unique point of user interaction are major assets for ensuring the acceptance of HA at the organizational level (bypassing the technical disparities across territories) and the individual level (reducing the demand on cognitive or learning resources for using assistive services).

Another study challenge is to provide 24/7 technology interventions requiring reliable internet access and good-quality bandwidth in areas offering disparate digital infrastructure. Technologically, any elaborate assistance service is based on the internet, and it is impossible to guarantee its continuous operation. To minimize service interruptions, we ensured the use of a single internet operator to avoid multiple service interruptions owing to operator-dependent updates. Covering a wide spectrum of needs and their evolution relies critically on the ability to populate the HA service catalog with a range of applications that match these needs. To do so, the HA leverages a dedicated integrated development environment while relying on needs analysis and human-centered design. This strategy is of paramount importance to ensure that the proposed assistive support is personalized to fulfill the needs of the participants and caregivers.

Despite these challenges, the outcomes of the HA study will yield significant insights into the benefits of AAL in frail older adults. It will also yield some insights into the factors influencing technology acceptance and use in FI. Indeed, it will be possible to explore how they vary according to participant characteristics, such as gender, age, cognitive abilities, and frailty level. The study will also inform the feasibility of technology-based personalization of multidomain assistive services and will probably teach some useful lessons for future technology-based field studies.

### Dissemination Plan

The results of this study will be disseminated through scientific publications and conferences. In addition, as we previously included in the HA design phases, the decision makers of the French public policy of assistance to the autonomy of older adults ensured that the solution is financially sustainable (<€ or US $1200 per home) by the public services so that a gain of only 1 month of aging in place produced by HA is more profitable than the monthly cost of a nursing home (>€ or US $1800 in France). This will provide a solid foundation for a business model when there is a start-up to market the technology.

### Conclusions and Future Directions

Although further research is needed, the findings of the HA project will provide the very first evidence-based study in IoT-based AAL in-home environments involving user-centered design for frail and nonfrail older adults.

To move forward with evidence robustness, it will be necessary to go further and consider conducting a trial using strict randomized controlled trial methods. Similarly, there is the question of scaling up for large-scale intervention. As also mentioned, our ecosystemic and participatory approach has led us to work directly with the key stakeholders in frailty and home care for older adults in each territory, to both design an upstream HA and evaluate it downstream. Consequently, scaling up other territories for the evaluation of HA will be required thereafter for better proof of the interest of an AAL system such as HA.

## Appendix

Multimedia Appendix 1 Health-related part of the assessment.

Multimedia Appendix 2 HomeAssist device part of the assessment.

## Abbreviations

3C: Three-City
AAL: ambient assisted living
ADL: activities of daily life
AMI: aging multidisciplinary investigation
AT: assistive technology
FI: frail individuals
HA: HomeAssist
IADL: instrumental activities of daily life
IoT: Internet-of-Things
MMSE: Mini-Mental Scale Evaluation
