# Supplementary material for: Effectiveness of an Ambient Assisted Living (HomeAssist) Platform for Supporting Aging in Place of Older Adults With Frailty: Protocol for a Quasi-Experimental Study
Source: JMIR Res Protoc. 2022 Oct 26;11(10):e33351. doi: 10.2196/33351 (PMC9647465; doi:10.2196/33351)
Supplement: Multimedia Appendix 1 [file resprot_v11i10e33351_app1.docx]

# Multimedia Appendix 1: Health-related part of Assessment

## Independent living capabilities

They are firstly evaluated by collecting the possible nursing home admission and with a questionnaire assessing each 6 months the critical events threating independent living such as the number of falls, the number and duration of hospitalization and the number of chronic diseases. Second, older adults’ everyday functioning is assessed by two scales: the Preferences for Routinization Scale (EPR, [55]) assesses the routinization of activities of daily life (ADL) and behaviors, i.e., the execution of behaviors or activities (e.g., sleep and meal schedules, organization of personal objects, social exchange) in the same rhythm or way over time; the routinization preferences are documented as a marker of age-related vulnerability [56]; the IADL scale [40], answered by both the participant and their informal caregiver assesses the older adults’ everyday functioning in instrumental ADL; We also chose to perform hetero-evaluations of older adults’ functional status, as frail older adults are shown to be sometimes not accurate in self-evaluating their everyday difficulties (e.g., [57]).

## Frailty ratings

They are based on i) nutritional status with Mini-Nutritional Assessment [58, 59] including the Body Mass Index (BMI) and the brachial and calf perimeters indices ;ii) The Short Physical Performance Battery (SPPB [60]) and the Timed up and Go test [61] and iii) Sensory abilities self-rating, particularly visual acuity and hearing. Additionally, for a better covering of multidimensionality of frailty, mental health (depression symptoms on the Center for Epidemiologic Studies-Depression CES-D scale [42], cognitive functioning (Mini-Mental State Evaluation-MMSE for cognitive status, [23], the Benton test [41], the Isaacs Set Test [43] and the Trail Making Test –TMT [61] for memory and executive assessment) and everyday cognitive difficulties self-rated or hetero-rated by the caregiver [62] (Table 1). The measures chosen were based on evidence indicating that these psychological abilities play a role in the causation and aggravation of the frailty [63, 64] and they are also related to adoption of technology [65]. Hence, these measures were included to examine if mental and cognitive health can predict HA uses and then mediated HA benefits across time.

## Older adults’ Self-perceived health

It is scored with two scales : first, the General Self-Efficacy Scale [66] rating the belief in one's competence to cope with a broad range of stressful or challenging demands and second, the Short Form-36 (SF-36) questionnaire assessing the health-related quality of life [67], that entails eight dimensions (physical functioning, physical limitations, body pain, general health, vitality, social functioning, limitations due to emotional problems, and mental health) and provides two summary scores, namely physical score and mental score. These two scores are also good proxies of the self-reported exhaustion dimension into frailty [68].

## Caregivers’ Self-perceived health

It is scored with two scales: first, the Short Form-36 (SF-36, [67]) questionnaire assessing the health-related quality of life, and second the Zarit Burden Inventory [69] (Zarit et al., 1980) assessing subjective caregiver burden that can be defined as the experience of “enduring stress and frustration” by those who care for individuals with reduced autonomy.

Table 1 briefly describes the assessment tools that are common to the HA and the control groups.

Table 1. Description of the assessment batteries.

| **Measure-related objective** | **Assessed construct** | **Name of Measure** | **Measure Description** |
| --- | --- | --- | --- |
| **Screening**  **measures** | **Survey screening** | Survey screening | Included questions related to their age, gender, wedding status, education,, experience with computers, living arranges, recruitment sources. |
| **Efficacy measures** | **Physical Frailty** | MNA [57] | *The MNA* assesses the nutritional status, especially diet, body mass index and unintentional weight loss. An MNA score < 24 identifies patients requiring a multidisciplinary geriatric intervention. When this latter is successful, then the  *Body Mass indices.* First, the Body Mass Index (BMI) was calculated according to the standard formula [BMI=mass (kg) / (height (m))^2^]. The BMI is scored from 0 to 3 with higher values indicating higher BMI values. Second, the brachial and calf perimeters are scored from 0 to 2 with higher values indicating higher lean mass values. Summed, the two indices provide a score from 0 to 5, with a higher score indicating a better body mass. |
|  |  | SPPB [59] | *Five Chair Stands* (lower body strength): The participant is asked to stand up from a chair five times without using their arms. The time is recorded and the test is scored from 4 - the participant takes less than 11.1 sec to complete the task to 0 – the participant is unable to perform task.  *Static Balance Testing* consists of three sorts of standing: side-by-side stand, semi-tandem stand and tandem stand; each of them scored from 4 – the participant holds the three standing positions for more than 10 sec; to 0 – the participant did not attempt any standing position.  *Gait Speed Test* corresponds to a timed 4-meter walk. It is scored from 4 – time is less than 4.82 sec – to 0 – the participant was unable to do the walk. |
|  |  | TGUG [60] | *Timed Get Up and Go Test* (agility and dynamic balance). This test consists of rising from a chair, walking three meters, turning around, walking back to the chair, and sitting down. Time in seconds to complete the task is recorded. The task is scored as followed: 1 – the task is completed in more than 30 sec, 2- the task is completed from 20 to 30 sec, and 3 - the task is completed in less than 10 sec (in this case, mobility is considered normal). |
|  |  | Sensory abilities [70] | *Vision/hearing impairments.* are assessed with a three-point 3 Likert-self-assessment type scale, ranging from 0 to 2 (where 0 corresponds to the highest sensory loss). So, sensory scale provided score ranged from 0 to 4 with higher scores indicating better sensory functions. |
|  | **Activities limitations in daily life** | Nursing home admission and critical events | *Nursing home rate*. During the follow-up, we register the Nursing home admission event for computing the rates Nursing home Admission at 12 months and optionally at 24 months.  *Critical events*. We collect each 6 months, the occurrence of falls, the number of hospitalized days and the number of chronic illness. |
|  |  | EPR [55] | *Preferences for Routinization Scale* (EPR) is the French analog of Routinization scale (Reich and Zautra, 1991). It is composed of 10 items with 5-point Likert scales assessing daily life habits and behaviors (e.g., sleep and meal schedules, organization of personal objects, social exchange) such as “I like to move and to change activities” or “I prefer to get up and to go to bed at the same time every day.” The degree of agreement with different sentences is rated and the total score is from 10 to 50, higher scores representing greater preferences for routinization. |
|  |  | **IADL scale**^a^**[**40**]** | *Self-assessed IADL scale.* is constituted by 24 items screening for difficulties regarding several ADL, based on a 5-point Likert scale, a higher score indicating a greater range of difficulties.  Hetero-evaluated IADL Scale is completed by the caregiver and the scoring is similar to that for self-assessed IADL scale. |
|  | **Self-perceived Health** | GSE test [67] | *General Sel-efficacy test*. is a 10 item questionnaire such as : ”Thanks to my resourcefulness, I know how to handle unforeseen situations,” and ”When I am confronted with a problem, I can usually find several solutions.” Scoring is done by adding the responses made to the 10 items. Possible responses are “not at all true”, “hardly true”, “moderately true”, and “exactly true”, yielding a total score between 10 and 40. |
|  |  | SF36 [68] | *Short 24 Form-36 (SF-36) questionnaire.* is used for assessing the health-related quality of life in the older participants. This questionnaire is self-administrated and consists of 36 items, covering eight dimensions (physical functioning, physical limitations, body pain, general health, vitality, social functioning, limitations due to emotional problems, and mental health) and provides two summary scores, namely physical score and mental score. |
|  | **Caregiver’s self-perceived Health** | ZBI [69] | *Zarit Burden Interview (ZBI).* is a 22-item self-administered questionnaire assessing burden associated with functional/behavioral impairments and home care context. The items have content validity and take into account common areas of concern such as health, finances, social life and interpersonal relations. |
|  |  | SF36[68] | *Short 24 Form-36 (SF-36) questionnaire.* is used for assessing the health-related quality of life in the caregivers. This questionnaire is self-administrated and consists of 36 items, covering eight dimensions (physical functioning, physical limitations, body pain, general health, vitality, social functioning, limitations due to emotional problems, and mental health) and provides two summary scores, namely physical score and mental score. |
|  | **Mental Health** | CES-D [42] | *Center for Epidemiologic Studies - Depression Scale* is used to assess depressive symptomatology in the older participants. It consisted in a 20-item scale with scores ranging from 0 to 60, a score above 17 was considered as indicating the presence of depressive symptomatology. |
|  | **Cognitive functioning** | MMSE [23] | *Mini-Mental State Examination* is used as an index of global cognitive performance. It comprises orientation in time and space, registration, calculation, recall and language items, with total score on the MMSE ranging from 0 to 30. |
|  |  | CDS [62] | *The Cognitive Difficulties Scale (CDS)* is a 38-item self-report measure of subjective complaints regarding immediate and delayed memory, attention, language, temporal orientation, and psychomotor abilities. |
|  |  | Cognitive Scales [41, 71, 61, 43, 72] | **•** *The* *Benton Visual Retention test* (BVRT) is used to assess visual memory, visual perception and/or visual construction. After the presentation for 10 seconds of a stimulus card displaying a geometric figure, subjects are asked to choose the initial figure among four possibilities; 15 figures are successively presented. The score ranges from 0 to 15.  **•** *The Free and Cued Selective Reminding Test* (FCSRT) is used to assess verbal memory. It provides a measure of memory under conditions that control attention and cognitive processing, in order to obtain an assessment of memory not confounded by normal age-related changes in cognition.  **•** *The Trail Making test (TMT)* is used to assess visual attention and task switching. In the Trail Making Test, Part A (TMT-A) the task is to connect randomly located circles with numbers (1–25) in numerical order as fast as possible. In the Trail Making Test, Part B (TMT-B) the task is to connect alternately circles with numbers (1–13) and letters (A–L) in their respective sequence as fast as possible.  • *The Isaac set test (IST)* is used to assess language skills and executive functions. It consists in generating words belonging to 4 semantic categories (cities, fruits, animals, and colors,10 words each) in 15, 30 and 60 seconds, total IST score ranging from 0 to 40.  **•** *The Stroop test* is used to assess working memory and attention. The test involves a control task for which participants have to name as quickly as possible the color of dots (Dot condition—Card 1), the color of the ink in which neutral words are printed (Word condition—Card 2), and the color of the ink in which color names are printed (Interference condition—Card 3). The increase in time taken to perform the latter task compared with the control task is the Stroop interference effect. For each condition, the completion time and the number of errors are compiled, and interferences scores are derived by calculating the ratio between the time required to name the color of the ink in the Word and the time required to name the color of dots in the Dot conditions (low interference), and the ratio between the time required to name the colors in the Interference and the Dot conditions (high interference). |

^a^Primary oucome
