# Supplementary material for: Effectiveness of an Ambient Assisted Living (HomeAssist) Platform for Supporting Aging in Place of Older Adults With Frailty: Protocol for a Quasi-Experimental Study
Source: JMIR Res Protoc. 2022 Oct 26;11(10):e33351. doi: 10.2196/33351 (PMC9647465; doi:10.2196/33351)
Supplement: Multimedia Appendix 2 [file resprot_v11i10e33351_app2.docx]

# Multimedia Appendix 2: HA device part of Assessment

As shown in **Table 2**, participants in HA condition completed a HA device follow-up battery covering the user’s HA needs and HA experience/perception to which are added HA-related measures.

## The user’s HA needs

They are scored with six scales including the assessment of self-determined behaviors, coping and compensation strategies, and risk profile of frailty.

The self-determined behaviors

They are assessed with the modified Arc's Self-Determination Scale [73] providing measures of autonomy, self-regulation, psychological empowerment and self-realization. This measure is motivated by our previous results from our pilot study revealing that the self-determination-based design (e.g., self-choice of assistive services) of HA system is a successful way to enhance the self-determined behaviors across time while increasing the positive experience of technology use [18].

The coping and compensation strategies

These strategies in everyday life setting are assessed with two scales: i) The PIC (Personality in Intellectual Aging Context, [74] comprises Locus of Control subscales which estimate the elderly's sense of control in performing everyday activities (e.g., “my problem solving ability depends on how healthy I am”) with three dimensions: Powerful others, Internal, Chance. Some findings revealed that older adults with an internal locus of control are more inclined to accept new technologies and to exhibit technology use (e.g., [74]); ii) The Memory compensation questionnaire *(*MCQ, [75]*)* measures individual differences in the tendency to select particular strategies and to overcome perceived or real cognitive losses, especially regarding everyday memory functioning. Precisely, this scale assesses three main compensation mechanisms and the individual’s awareness for these mechanisms as defined in compensation theory [76]: 1) the substitution mechanisms which is replacing a declining technique by a new one or doing something in a different way; 2) the remediation mechanisms which require investing more time and effort to adapt and overcome losses; 3) the accommodation strategies which aim to reduce the mismatch between environmental demands and personal skills by adjusting one's goal; and 4) the personal insight and the beliefs regarding memory losses referring to the extent to which the individual is aware of changes in the compensation’s needs (i.e. external, internal, reliance, time and effort) over the 5–10 years prior to testing. Overall, MCQ provides a good approximate of need of external supports such as HA system enable to do through multiple forms of activity or goal –related reminders.

Frailty-related risk profile

It was assessed using three scales. First, the SEGAm (modified Short Emergency Geriatric Assessment) instrument validated for use among community-dwelling subjects [24, 78]. This instrument establishes a risk profile of frailty and provides reporting of problems and factors that may influence functional decline, including age, social environment, drugs, mood, perceived health, history of falls, nutrition, comorbidities, IADL, mobility, continence, feeding and cognitive functions. Second, there are some evidence showing that frailty benefits from sleep quality improvement along advanced ages, particularly the interventions over circadian rhythm [79]. As HA system can provide external support for good sleep habits, the quality of sleep is measured with the Pittsburgh Sleep Quality Index (PSQI, [80]) which rates subjective sleep quality, sleep latency, sleep duration, habitual sleep efficiency, sleep disturbances, use of sleeping medication and daytime dysfunction. Finally, social isolation or loneliness being also reported as risk factor of frailty [81], we also explored social networks, using the Lubben Social Network Scale [82] which assesses structural (e.g., network size), interactional (e.g., contact frequency) and functional components (e.g., purpose of support) of an individual’s family and friendship ties and was specially developed for older adults [83].

## The user’s HA experience

It is assessed with three scales assessing user experience, user satisfaction, and HA usefulness for family caregiver, respectively. The Attrackdiff questionnaire [84] decomposes user experience into five dimensions: ergonomic quality, hedonic quality, appealingness, anxiety and safety perception, and social influence. Second, the QUEST 2.0 questionnaire [85] measures user satisfaction with assistive technologies with two components, “Device” (hardware aspects) and “Services” (software aspects). The third scale is the IADL support scale which is an adaptation of the Lawton scale presented earlier. This adaptation aims to assess the HA usefulness for the family caregiver in terms of burden for IADL support.

## The HA-related measures

They deliver three categories of indicators regarding participant in their living space. First, passive interactions with HA provide actimetric data. The actimetric data quantify at the beginning of the experiment, and every 6 months, the level of activity in the home (quantity and duration of daytime and night activity), where this activity takes place in the home (bedroom, bathroom, living room and kitchen), the approximate level of nutrition-related activity with a specific monitoring of fridge opening and closing and the level of activities out of home (approximated by the number of exits from the home each day and their duration).

HA uses and usages are assessed thanks to active interactions with the HA platform, and more particularly with the primary and secondary tablets. The use behaviors of HA assistive services are quantified through the received notifications (critical and non-critical), the participant answers to these notifications, and the system pause occurrences. Such measures make it possible to objectify the intensity of the support provided by the platform but also its invasive or inappropriate interactions (pause of the system by the user). The uses and usages of the secondary tablet are quantified in terms of use diversity (number of opening apps) and associated usages according to the classification by Olson et al. in 2011 [86], i.e., “internet search”, “communication”, “financial management”, “health services”, “home equipment”; “leisure”, “shopping” and “transport”. The indicators were collected across a period of 6 months.

Table 2 describes all the HA-related measures for the HA group

Table 2: Description of the additional assessment batteries for HA condition.

| **Measure-related objective** | **Assessed construct** | **Name of Measure** | **Measure Description** |
| --- | --- | --- | --- |
| **User’s HA needs** | **Motivation, Coping and compensation strategies** | Arc's Self-Determination Scale [73] | *The Arc's Self-Determination Scale*. is a 72-item scale which assesses self-determined behaviors. It is composed of four dimensions: the first one measures autonomy (32 items), the second measures self-regulation (9 items), the third dimension assesses psychological empowerment (15 items) and the last dimension measures self-realization including self-awareness and self-knowledge (14 items). Seventeen items of this scale are excluded because of their inappropriateness with respect to the topic of our study (in particular, items related to working conditions) ; the 55-selected items can be found in [19]The final scale is composed by 55 items, including 26 items for measuring autonomy, 4 items for self-regulation, 10 items for empowerment and 15 items for self-realization. The items are scored the same way as the original Arc's scale, where a higher score indicates a better self-determination performance. Participants underwent this questionnaire tests twice times, i.e. before the HA installation (baseline measurement), and at 12 months after the HA installation, and optionally at 24 months after the HA installation. |
|  |  | PIC [74] | *The Personality in Intellectual Aging Context (PIC) scale*. provides Locus of Control subscales. These scales consist of 12 items and estimate the elderly's sense of control in performing everyday activities (e.g., “my problem solving ability depends on how healthy I am”) with three dimensions: Powerful others, Internal, Chance. Responses are scored on a 6-point Lickert scale ranging from 6 (strongly agree) to 1 (strongly disagree). Each dimension is scored from 12 to 72, higher scores indicating greater beliefs in internal/chance/other control of one's capabilities. Participants underwent this scale twice times, i.e. before the HA installation (baseline assessment), and at 12 months after the HA installation, and optionaly at 24 months after the HA installation. |
|  |  | MCQ[75] | *The Memory compensation questionnaire.* is a seven-factor scale measuring individual differences in the tendency to select particular strategies and to overcome perceived or real memory losses. (F1) The External scale contains eight items regarding the use of external aids and devices for supporting remembering (e.g., shopping lists, notes, putting things in a specific place). (F2) The Internal scale contains ten items regarding the use of strategic mnemonic strategies to facilitate or improve memory efficiency. (F3) The Reliance (or Recruitment) scale contains five items related to the recruitment of other people for memory assistance. These first three scales relate to the Substitution mechanism of compensation theory [76]), which is replacing a declining technique by a new one or doing something in a different way. (F4) The Time scale contains four items regarding the extent to which the respondent invests more time in performing memory tasks. (F5) The Effort scale contains six items regarding the investment of more effort when performing a memory task such as rehearsing or retrieving information. The Time and Effort scales relate to Remediation mechanisms which require investing more time and effort to adapt and overcome losses. Finally, two scales investigate general aspect of memory compensation. (FG1) The Success scale contains five items regarding the use of Accommodation strategies which aim to reduce the mismatch between environmental demands and personal skills by adjusting one's goal. It assesses the extent of commitment to memory performance and the motivation to maintain a given memory competence. The higher the commitment, the less a person will tend to accommodate to his/her losses as he/she will maintain the same criterion of success and sense of control. Finally, (FG2) the Change scale contains five items regarding the extent to which the respondent is aware of changes in the need of compensation (i.e. external, internal, reliance, time and effort) over the 5–10 years prior to testing. This last scale reflects personal insight and beliefs regarding memory losses. Participants respond to each item (except for the change scale) on a 5-point scale (0 =*never*, 1 = *seldom*, 2 = *sometimes*, 3 = *often*, and 4 = *always*.) The Change scale has the following scale: 0 =*much less often*, 1 = *less often*, 2 = *no difference*, 3 = *more often*, and 4 = *much more often*; except for the item “Do you spend more or less time learning important things today compared with 5–10 years ago (e.g., reading things more slowly or reading them more than once” which had the choice options: 0 =*much less time*, 1 = *less time*, 2 = *no difference*, 3 = *more time*, and 4 = *much more time*)*.*  This scale is administrated twice times, i.e. before the HA installation (baseline assessment), and at 12 months after the HA installation, and optionally at 24 months after the HA installation. |
|  | **Risk profile of Frailty** | SEGAm scale [24, 78] | *The SEGAm instrument* (modified Short Emergency Geriatric Assessment) assesses multi-dimensionally the frailty. It is composed of two parts (A and B). The part A comprises 13 items (age, living space, medication, mood, perception of own health, fall in the last 6 months, nutrition, associated diseases, IADL, mobility, continence, meals, cognitive function, especially orientation and memory), coded from 0 (most favorable situation) to 2 (least favorable situation), giving an overall score of 26 points. Subjects are considered as not very frail (score <8), frail (score between 8 and 12), or very frail (score >12).  The part B called « complementary data » comprises 11 items [hospitalization in the last 6 months, vision, audition, family support, home service, natural caregiver need, (natural) caregiver burden perception, subject’s life project, life project according to caregiver), ), coded from 0 (most favorable situation) to 2 (least favorable situation), giving an overall score of 22 points ; the higher the score, the greater the fragility.  This scale is administrated three times, i.e. before the HA installation (baseline assessment), and at 6 and 12 months after the HA installation, and optionally at 18 and 24 months after the HA installation |
|  |  | PSQI [80] | *The Pittsburgh Sleep Quality Index (PSQI).* is a self-rated 19 item-questionnaire which assesses sleep quality and disturbances over a 1-month time interval. It provides 7 component scores, i.e., subjective sleep quality, sleep latency, sleep duration, habitual sleep efficiency, sleep disturbances, use of sleeping medication and daytime dysfunction, each weighted equally on a 0-3 scale. The 7 component scores are summed for providing a global PSQI score ranging from 0 to 21; higher score indicate worse sleep quality. Participants underwent this questionnaire tests twice times, i.e. before the HA installation (baseline measurement), and at 12 months after the HA installation, and optionally at 24 months after the HA installation. |
|  |  | LSN Scale [82,83] | *Lubben Social Network (LSN) Scale.* assesses isolation by providing an assessment of person’s social connections in terms of both family interactions and interactions with friends. Precisely, this scale assesses structural (e.g., network size), interactional (e.g., frequency of contact) and functional components (e.g., purpose of support) of an individual’s family and friendship ties. Scores range from 0 and 60, with a higher score indicating more social engagement and better networks.  This scale is administrated twice times, i.e. before the HA installation (baseline assessment), and at 12 months after the HA installation, and optionally at 24 months after the HA installation |
| **HA perceptions** | **User Satisfaction** | QUEST 2.0 [85] | *The QUEST 2.0 questionnaire.* is a measure of user satisfaction with assistive technologies with two components, Device and Services. The questionnaire consists of 12 items and each item is evaluated on a scale ranging from 1 to 5. A value 1 corresponds to ‘not satisfied at all’ and a value 5 to ‘very satisfied’. The total score is on a scale of 5. A high score denotes a high user satisfaction with the technology. HA Participants underwent this questionnaire twice (6 and 12 months after the HA installation) and optionally two additional times, i.e. 18 and 24 months after the HA installation. |
|  | **User experience** | Attrakdiff questionnaire [84] | *The Attrakdiff questionnaire.* decomposes user experience into five dimensions: ergonomic quality, hedonic quality, appealingness, anxiety and safety perception, and social influence. Answers to the questionnaire range over a scale of 7 points, from −3 to 3, including two antonyms (e.g., nervous/relaxed). Each dimension of the questionnaire consists of 6 items. Participants underwent this questionnaire tests three times, i.e. before the HA installation (baseline for participant’s Perceptions regarding Assistive technologies), at 6 and 12 months after the HA installation, and optionally at 18 and 24 months after the HA installation. |
|  | **Caregiver’s usefulness perception** | IADLSupport scale [19] | *The IADL support scale.* is an adaptation of the Lawton scale presented earlier, to assess burden for IADL support. Answers varied from 0 (very easy to assist), to 4 (very hard to assist), in reference to the assistance given to the participant in particular. For instance, an item of this scale is: “For you, the  support that you provide for eating is: Very hard–Very easy.”  Caregivers underwent this questionnaire tests two times, i.e. before the HA installation (baseline for participant’s Perceptions regarding Assistive technologies) and 12 months after the HA installation, and optionally at 18 and 24 months after the HA installation. |
| **HA-related measures** | **Actimetric data** | Level of Activity at home | *Level of activity at home.*  is measured with three indicators: 1) Quantity and duration of daytime activity; 2) Quantity and Duration of night activity and then 3) Cumulative activity.  Quantity and Duration of activity are approximated by averaging all movements (detected and registered by HA system) over a period of 30 consecutive days starting at the time of installation, then 6 months and 12 months after installation (optionally at 18 and 24 months). |
|  |  | Specific located activities | *Specific located activities* refer to movement activity located in a specific room of the home, namely bedroom, bathroom, living room, and kitchen. Nutrition-related activities are specifically approximated by the detection of opening/closing of fridge each day. |
|  |  | Level of Activities out of home | *Level of Activities out of home* is approximated by the number of exits from the home each day and their duration. |
|  | **HA Use and Usage** | Primary tablet-based Assistive services Uses | *The use* of *HA assistive services* is quantified by the following measures across periods of 6 months:  -Total number of notifications on the primary Tablet then separately the number of non-critical and critical notifications  -Number of responses to critical notifications  -Number of consultation of notifications  -Number of pauses of HA system |
|  |  | Secondary tablet-based uses and usages | *The uses and usages of the secondary tablet* are quantified thanks to the following indicators across periods of 6 months:  - Number of browser openings  - Number of openings of simplified mailer  - Nb of mail received vs. nb of mail sent  - Number of applications opened  - Number of opening app according to the need targeted classification by Olson et al., 2011, i.e., “communication”, “financial management”, “health services”, “home equipment”; “leisure”, “shopping” and “transport”. |
